# Correlation between Degenerative Thoracolumbar Kyphosis and Lumbar Posterior Muscle

**DOI:** 10.3390/jpm13101503

**Published:** 2023-10-16

**Authors:** Chen Guo, Shuai Xu, Yan Liang, Bin Zheng, Zhenqi Zhu, Haiying Liu

**Affiliations:** Department of Spinal Surgery, Peking University People’s Hospital, Peking University, Beijing 100044, China; fantasy_g@pku.edu.cn (C.G.); xushuairmyy@pku.edu.cn (S.X.); liangyan7503@163.com (Y.L.); zhengbin97@163.com (B.Z.); zhenqizhu@sina.com (Z.Z.)

**Keywords:** degenerative thoracolumbar kyphosis, lumbar stenosis syndrome, body mass index, paraspinal muscle, lumbar crossing indentation value

## Abstract

The relationship between spinal alignment, particularly degenerative thoracolumbar kyphosis (DTLK) combined with lumbar spine stenosis (LSS), and paraspinal muscle content remains underexplored. This study aimed to elucidate the characteristics of paraspinal muscle distribution in DTLK patients and its association with lumbar lordosis (LL) and body mass index (BMI). Methods: A case–control study was conducted comparing 126 patients with DTLK and LSS against 87 control patients. The lumbar crossing indentation value (LCIV) was introduced as a novel measurement for paraspinal muscle content, and its relationship with thoracolumbar kyphosis (TLK), BMI, and LL was assessed. Results: LCIV in DTLK patients was found to be lower than in the control group, with a progressive increase from the upper to lower lumbar spine. In the control group, paraspinal muscle content was observed to increase with age and BMI, and LCIV was higher in males. However, the DTLK group showed no gender difference. LCIV in the DTLK group was more pronounced in patients with increased LL. The degree of TLK was not influenced by BMI but was associated with the content of the paravertebral muscle. Conclusions: Paraspinal muscle content, as measured by LCIV, is significantly associated with DTLK and LSS. The study emphasizes the importance of considering paraspinal muscle health in DTLK patients and offers valuable insights for diagnosis and therapeutic interventions.

## 1. Introduction

Adult spinal deformity (ASD) encompasses a range of pathological conditions that disturb the spinal alignment, affecting both the young and old. Degenerative thoracolumbar kyphosis (DTLK), a subclass of adult spinal deformity (ASD), is a prevalent degenerative spinal disease in the elderly, particularly those over 50 years of age [1,2]. This age group was specifically targeted in our study due to the higher incidence and exposure rate of DTLK combined with lumbar stenosis syndrome (LSS) in middle to older age groups. As the global population ages, the prevalence of DTLK, often co-occurring with lumbar spinal stenosis (LSS), is expected to rise, making it an increasingly significant public health concern. The condition can lead to low back pain, lower limb radiation pain, and intermittent claudication, significantly affecting the quality of life [3]. There are age and gender related differences in the severity of DTLK [4,5]. Interestingly, the relationship between body mass index (BMI) and DTLK remains ambiguous. While some studies have found a correlation between obesity and spinal degeneration, others have emphasized that muscle content, rather than BMI, correlates with the degree of deformity [1]. This inconsistency in the literature underscores the need for further investigation. Paraspinal muscle groups, such as the multifidus and erector spinae, play a crucial role in maintaining normal spine formation, and their weakening is closely related to the severity of deformity [6,7,8]. Various methods to measure the paravertebral muscle component have been employed. The most common indicators are muscle cross sectional area (CSA) and fat infiltration (FI) by MRI [9,10,11], but it is labor-intensive and complicated, limiting their applicability in clinical settings. To address this limitation, the concept of lumbar crossing indentation value (LCIV) was proposed by Takayama et al. [12], offering a simplified yet robust way to measure paravertebral muscle characteristics, making it an attractive metric for broader clinical adoption. While LCIV has been described in the context of the elderly without spinal deformities, its role in patients with DTLK has not yet been elucidated. The aim of this study was twofold: firstly, to explore the paraspinal muscle characteristics in patients suffering from DTLK co-occurring with LSS, and secondly, to elucidate any correlations that may exist between thoracolumbar kyphosis (TLK) degree, BMI, and LCIV. Understanding these relationships holds pivotal implications for both diagnostic strategies and treatment modalities in DTLK. Moreover, our research introduces LCIV as a convenient and cost-effective metric that could be easily implemented in an outpatient setting or even self-administered by patients for initial diagnosis. By focusing on patients aged 50 years and above, we aimed to tailor our findings towards a demographic that is disproportionately affected by DTLK and LSS, thereby facilitating more personalized and effective clinical interventions.

## 2. Materials and Methods

### 2.1. Participants

The single-center retrospective study was conducted from June 2015 to December 2020. Eligible participants from our institution diagnosed with LSS and DTLK requiring surgical intervention were enrolled in the DTLK group, while those with simple LSS requiring surgery served as the control group. The study received ethical approval from our institution, and informed consent was obtained from all participants.

The inclusion criteria were: (1) patients in the DTLK group diagnosed as DTLK with LSS without coronal malformation, (2) control group patients with simple LSS, (3) complete and clear preoperative X-ray of the whole standing spine and thoracolumbar or lumbar MRI, (4) degenerative spine disease (age > 50 years). The age cutoff of 50 years was chosen because previous research has shown that LCIV remains stable in individuals over this age [12].

**Figure 1:**
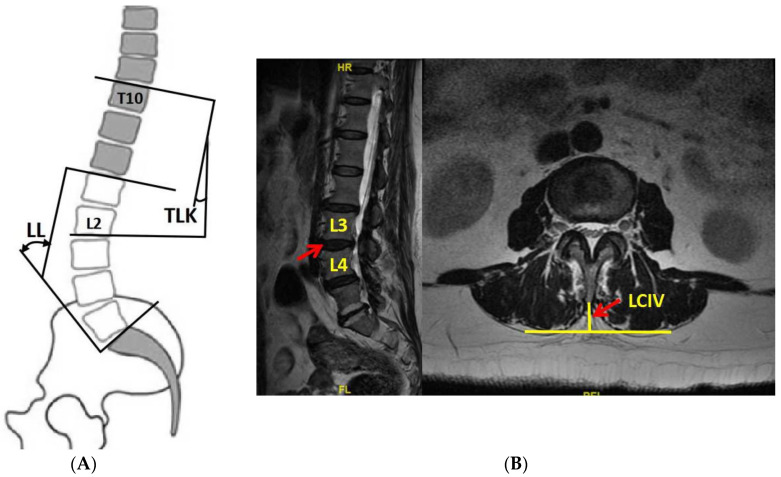
(**A**) The definition of TLK and LL; (**B**) the definition of LCIV. TLK: thoracolumbar kyphosis; LL: lumbar lordosis; LCIV: lumbar crossing indentation value.

Exclusion criteria were: (1) coronary imbalance or abnormalities; (2) incomplete or blurred imaging of X-ray or MRI; (3) diagnosis of idiopathic scoliosis, congenital scoliosis, or other spinal deformity; (4) patients with lumbar spine fracture, tumor, infection, or spondylolisthesis; (5) those who had undergone thoracolumbar surgery. Coronal scoliosis is Cobb angle > 10° and imbalance is defined as sagittal vertical axis (SVA) > 3 cm. Kyphosis severity is expressed by TLK (the angle between the upper end plate of T10 and the lower end plate of L2) and DTLK is TLK ≥ 15° caused by degeneration (Figure 1A).

### 2.2. BMI

BMI equals weight (kg) divided by the square of height (m^2^). Patients were classified into normal weight (N), overweight (OW), and obesity (OB) according to BMI where 18.5 ≤ N < 25.0 (kg/m^2^), 25.0 ≤ OW < 30.0 (kg/m^2^), and OW ≥ 30.0 (kg/m^2^).

### 2.3. MRI

All subjects underwent MRI in a supine position using a 1.5-T scanner (Gyroscan; Philips Medical Systems, Washington, DC, USA). Axial 5 mm slices were acquired using three-dimensional thick T2-weighted spin-echo axial scanning (TR: 3200 ms; TE: 102 ms; FOV: 180 mm; matrix size: 512 × 512) from T12 to L5. The sagittal and axial images were uploaded to the PACS client software (Easy Vision IDS5, version 11.4; Philips, Hamburg, Germany). The axial images were obtained from the intervertebral space parallel to endplates.

### 2.4. Parameters Measurement

TLK and lumbar lordosis (LL) were measured on whole-spine X-ray. LL is the angle from upper endplate of L1 to upper endplate of S1 (25° ≤ LL < 53°). T2 axial images were used for LCIV measurement (vertical distance from spinous process to the apex of paravertebral muscle on both sides (Figure 1B)). LCIV from T12-L1 to L4-L5 was separately measured, while L5-S1 was excluded due to the huge heterogeneity from occlusion of iliac crest and iliopsoas muscle attachment.

All parameters were measured by two persons independently and consistency was evaluated by intraclass correlation coefficient (ICC). ICC of TLK and LL were 0.86 and 0.79; ICC of each segment of LCIV was 0.90 (T12-L1), 0.78 (L1-L2), 0.78 (L2-L3), 0.83 (L3-L4), and 0.77 (L4-L5), which indicates all measurements showed good reliability.

### 2.5. Subgroup Analysis

A subgroup analysis of gender (male, female) and BMI (N, OW, and OB) was performed to determine whether gender and BMI have an effect on LCIV. Subgroup analysis was also performed for DTLK patients according to LL with larger LL group (LL ≥ 53°), normal LL group (25° ≤ LL < 53°), and less LL group (LL < 25°).

### 2.6. Statistical Analysis

Dichotomous variables between groups were tested by χ^2^ test or Fisher test. Independent-sample t-test was used for inter-group comparisons of measurement data, while analysis of variance (ANOVA) analysis was used for comparisons of multiple measurements in the same group. These were also applied for comparison among subgroups. Pearson correlation analysis was used for correlation evaluation among TLK, BMI, and LCIV, and multiple linear regression was used to determine the influencing factors of LCIV. The software SPSS 22.0 (IBMC, Armonk, New York, NY, USA) was used for statistical analysis, and statistical differences were determined with *p* < 0.05.

## 3. Results

A total of 126 patients were included in the DTLK group, and 87 cases were in the control group. There were no statistical differences between the two groups in terms of gender, age, and BMI (*p* > 0.05). The TLK in the DTLK group was 25.8° ± 10.1° (ranging from 15.2° to 64.2°) (Table 1).

### 3.1. LCIV in DTLK and Control Groups

The LCIV in the DTLK group was less than that of the control group in the segments T12-L1, L1-L2, and L2-L3 (*p* < 0.01), but not in the lower lumbar spine. The mean LCIV (mLCIV) of the DTLK group was also less than that of the control group (*p* < 0.01). The LCIV showed an increasing trend in the DTLK group from T12-L1 to L4-L5, with significant intra-group differences (*p* < 0.01) (Table 2).

There were no gender differences in TLK in the control group (*p* = 0.924). The mLCIV and LCIV for all segments in males were larger than in the female group (*p* < 0.05), except at the T12-L1 level. In the DTLK group, there were no gender-related differences in all segments and mLCIV, except for the T12-L1 segment (*p* = 0.044) (Table 3).

The TLK showed no differences with various BMIs in both groups (*p* = 0.605 and *p* = 0.464). In the control group, both LCIV and mLCIV differed among the three subgroups by BMI (*p* < 0.05), where LCIV in the N subgroup was less than the OW subgroup from T12-L1 to L3-L4 (*p* < 0.05), and less than the OB subgroup at all levels (*p* < 0.01). In the DTLK group, LCIV values at T12-L1 and L1-L2 were significant with various BMIs (*p* = 0.003 and *p* = 0.009). Although mLCIV in the N subgroup was smaller than the OB subgroup, there was no statistical difference in mLCIV among subgroups (*p* = 0.080) (Table 4).

**Figure 2:**
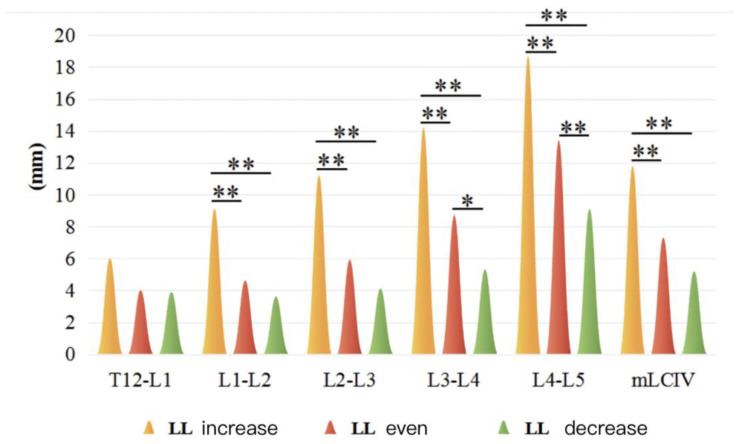
LCIV in increased LL and decreased LL subgroup in DTLK group. *: statistical difference between variables (*p* < 0.05); **: statistical difference between variables (*p* < 0.01).

**Figure 3:**
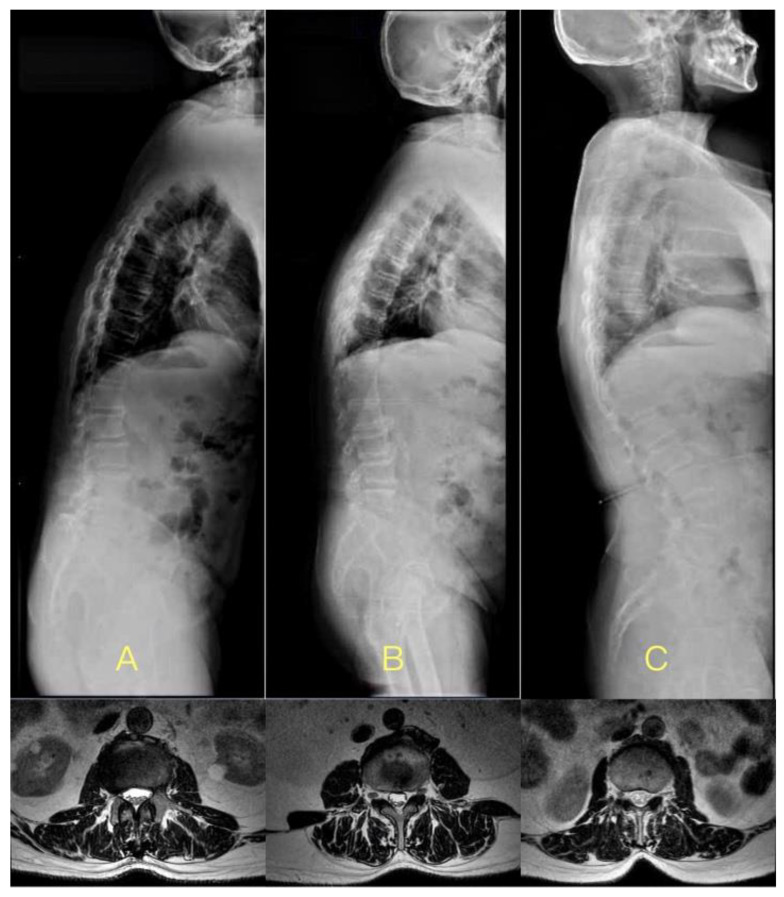
The cases with DTLK in less LL, normal LL, and larger LL subgroups, respectively. (**A**) 73-year-old male, BMI = 26.4 kg/m^2^, TLK = 26.3°, LL = 5.9°, LCIV in L3–4 was 7.2 mm, mLCIV was 5.8 mm; (**B**) 68-year-old male, BMI = 26.5 kg/m^2^, TLK = 28.2°, LL = 32.1°, LCIV in L3–4 was 11.2 mm, mLCIV was 7.5 mm; (**C**) 68-year-old male, BMI = 25.8 kg/m^2^, TLK = 29.8°, LL = 61.9°, LCIV in L3–4 was 5.2 mm, mLCIV was 12.0 mm.

There were 26 cases, 64 cases, and 36 cases in the larger, normal, and less LL subgroups, respectively, in DTLK patients. Both LCIV and mLCIV were significant among the three subgroups (*p* < 0.01), except for the T12-L1 segment. LCIV from L1-L2 to L4-L5 and mLCIV in the increased LL subgroup were larger than those of the normal LL and less LL subgroups (*p* < 0.01) (Figure 2 and Figure 3).

### 3.2. The Influencing Factor of LCIV

In the control group, mLCIV positively correlated with BMI (r = 0.328, *p* = 0.004) but not with TLK. In the DTLK group, TLK showed a negative correlation with mLCIV (r = −0.480, *p* < 0.001) but not with BMI. The integration of both groups showed mLCIV positively correlated with BMI (r = 0.201, *p* = 0.009), while negatively with TLK (r = −0.397, *p* < 0.001).

mLCIV was seen as the dependent variable, and other variables (*p* < 0.1) were considered independent variables. In the control group, the independent influencing factor was BMI (*p* < 0.01), with mLCIV (mm) = 0.33 × BMI (kg/m^2^) (0 ≤ TLK < 15°). In the DTLK group, the independent influencing factor was TLK (*p* < 0.001), and mLCIV (mm) = 13.75 − 0.48 × TLK (°) (TLK ≥ 15°). The integration of the two groups showed that mLCIV was determined by both TLK and BMI, where mLCIV (mm) = 5.41 + 0.22 × BMI (kg/m^2^) − 0.41 × TLK (°) (Table 5).

## 4. Discussion

The significance of sustaining spinal alignment and sagittal balance has received increasing attention, with key contributions pointing toward the integral role of the paraspinal muscle groups, particularly the multifidus and erector spinae muscles [6,7,13]. These muscles serve not only as dynamic stabilizers, but also as key players in maintaining spinal biomechanics, thereby preventing or slowing down deformities and functional impairments. Yagi et al. [7] explored this in their groundbreaking multicenter retrospective study involving 60 patients with adult spinal deformity (ASD). Utilizing advanced imaging techniques, they found a marked correlation between the cross-sectional area (CSA) of the multifidus and erector spinae muscles and the severity of sagittal spinal disorders. This significant finding underscores the complex and bidirectional relationship between spinal alignment and the functional integrity of paravertebral muscles. Furthermore, Yagi et al. suggested that age is a key modifier in this relationship. In older populations, where muscle atrophy is more prevalent, the decline in paraspinal muscle CSA could exacerbate existing spinal deformities [13,14]. Adding to this narrative, Mannion et al. [15] conducted a nuanced analysis using histochemical methods to explore the tissue characteristics of paravertebral muscles in ASD patients. They discovered that degeneration of these muscles was not merely a coincidental feature but appeared to be a secondary progression strongly associated with the severity of spinal deformities, such as scoliosis. Their work introduced the concept that the biochemical milieu within these muscles, potentially altered due to prolonged mechanical stress, could contribute to the progressive nature of spinal deformities. The work of both Yagi et al. and Mannion et al. collectively highlights the intertwined relationship between muscle health and spinal integrity. These studies establish a compelling case for considering paravertebral muscle status as a key parameter during both the diagnostic phase and treatment planning for spinal deformities.

Banno et al. [7] observed that in ASD patients, abnormalities in sagittal parameters and the lumbar spine were associated with the CSA of multifidus, even after adjusting for weight and age. However, the muscle content in patients with thoracolumbar deformity has been largely overlooked. Given the increasing prevalence of DTLK with LSS in middle-aged to elderly populations, understanding the relationship between TLK and paravertebral muscles is crucial [16]. Traditional methods for measuring paravertebral muscles are intricate and time-intensive, making them impractical for outpatient settings. The introduction of the LCIV concept by Takayama et al. streamlined this process, making it more efficient and widely applicable [12]. While some studies suggest that obesity exacerbates degenerative scoliosis, others argue that muscle composition, rather than obesity, influences sagittal alignment [1,17]. Our findings showed that individuals with a higher BMI had greater muscle content, but body shape did not influence TLK in the control group. In the DTLK group, the degree of TLK was not associated with BMI, but was related to paraspinal muscle content.

Takayama et al. [12] laid important groundwork in the field by establishing a robust correlation between lumbar crossing indentation value (LCIV) and cross-sectional area (CSA) across all lumbar segments, with a Pearson’s correlation coefficient ranging from 0.708 to 0.789. This significant correlation was groundbreaking as it validated LCIV as an efficient, alternative metric for evaluating paraspinal muscle content, especially beneficial in outpatient and low-resource settings. They also identified a negative correlation between LCIV and age, although this correlation lost its statistical significance among individuals aged 50 and above. This observation led us to question how LCIV behaves in a specialized cohort, such as patients with degenerative thoracolumbar kyphosis (DTLK). Our study, in contrast to the general trends reported by Takayama et al., found a noticeable reduction in LCIV values within the DTLK patient group when compared to the control cohort. One compelling hypothesis for this phenomenon revolves around anatomical changes in the spinal structure inherent to DTLK. Specifically, the approximation of the spinous processes to the skin—caused by local kyphosis in the thoracolumbar and upper lumbar spine regions—appears to significantly influence LCIV measurements.

Concurrently, our observations indicate that the posterior muscle groups are subject to thinning in DTLK patients. This is likely a biomechanical response to the pressure exerted by the altered, kyphotic bone structure, which would naturally decrease the available space for muscle tissue. These findings provide further credence to the notion that the CSA of the paraspinal muscle is intricately linked with progressive spinal kyphosis [9,18]. Of particular note was the localization of these changes. The most pronounced differences in LCIV between our study groups were predominantly observed in the upper lumbar spine. The lower lumbar regions, conversely, displayed relatively comparable LCIV values between the groups. This may suggest that the impact of DTLK on paraspinal muscle content is not uniformly distributed across the lumbar spine, but is more concentrated in specific regions, warranting further investigation.

Alterations in lumbar lordosis (LL) can lead to compensatory changes, such as pelvic rotation and proximal thoracolumbar kyphosis [19,20]. In our study, 20.6% of patients exhibited increased LL, while 28.6% showed decreased LL. Those with a pronounced LL had deeper skin grooves and more prominent posterior muscles. Conversely, patients with reduced LL had diminished paraspinal muscle content due to spinal kyphosis and limited muscle attachment space. This was further exacerbated by the weakened strength of the lower back muscles in these patients [7]. Previous studies have also highlighted the relationship between posterior muscle strength and LL loss or kyphosis in older individuals [18]. Thus, interventions aimed at restoring LL, either surgically or conservatively, can enhance muscle volume and distribution. This underscores the significance of strengthening back muscles to maintain or improve spinal alignment.

Gender differences in muscle density and CSA are evident in the general population [4,21]; consistently, studies have shown that males exhibit greater muscle density and CSA compared to their female counterparts, potentially due to hormonal differences that influence muscle growth and maintenance. Our findings on LCIV corroborate this observation. However, this gender-based trend in LCIV measurements deviated when examining the DTLK patient cohort. Contrary to expectations, the difference in muscle content between males and females in this group was statistically insignificant. One plausible explanation could be age-related muscle atrophy, a phenomenon well-documented in geriatric populations. The progressive decline in muscle mass and density due to aging appears to negate the usual gender-related advantages seen in younger populations. Thus, our findings suggest that while gender differences in muscle content are pronounced in the general population, these differences become attenuated or even nullified in the context of DTLK. This is a significant observation, indicating that diagnostic and treatment protocols for DTLK need to be gender-neutral. It becomes crucial, therefore, to consider muscle content and composition in a more nuanced manner, focusing on individual patient profiles rather than making assumptions based on gender alone. Our study reinforces the need for further investigations into how gender-specific changes in muscle content evolve with age and how these changes interact with spinal deformities such as DTLK. Such insights will be indispensable for tailoring more effective therapeutic interventions.

Obesity exerts additional load on the spine, leading to a reduction in intervertebral space height and diminished disc shock absorption capacity. Chronic spinal compression due to obesity can trigger inflammation through the release of adipocyte factors, further exacerbating spinal degeneration [22,23]. While some studies link BMI to the development and progression of degenerative scoliosis [3], others, such as Kim et al. [24], offer a more nuanced perspective. In our study, TLK was not influenced by BMI in either the DTLK or control groups, aligning with Kim et al.’s findings. Interestingly, the muscle content in the control group was significantly different between the normal and obese subgroups. This suggests that obese individuals, despite their larger stature, also have denser muscles, potentially to support the additional weight [17]. In the DTLK group, muscle content was more influenced by kyphosis than BMI. Moreover, muscle content and strength were reduced in DTLK patients, especially those with higher BMIs [25], a finding supported by MRI and intraoperative dissection [26].

This study is the first to elucidate the relationship between BMI and paraspinal muscle distribution in DTLK patients using a case–control approach. It underscores the need to prioritize paraspinal muscle health in DTLK patients and highlights the benefits of post-surgical functional exercises or rehabilitation training to restore sagittal spinal alignment [27]. The introduction of LCIV has revolutionized the measurement of paraspinal muscles, making it more accessible and cost-effective for both clinicians and patients [12,28]. Moreover, our research quantified the relationship between TLK, BMI, and LCIV, providing valuable insights for primary diagnosis and therapeutic interventions for DTLK. However, our study has several limitations. Firstly, the LCIV measurement, although efficient, may not offer the same level of precision as CSA, particularly for muscle tissues with significant fatty infiltration. Secondly, our cohort includes a broad age range (50–87 years) without stratification; despite LCIV measurements being stable after age 50, this could potentially introduce reporting bias. Thirdly, our findings specifically pertain to DTLK patients with LSS and may not generalize to other spinal deformities, such as ankylosing spondylitis, Scheuermann’s disease, or coronal scoliosis. Fourthly, the study’s retrospective design means our conclusions would benefit from validation through a prospective cohort with a larger sample size. Lastly, we did not account for occupation and physical activity levels of the participants, factors that could significantly impact paraspinal muscle health. This omission could be particularly relevant given the advanced age of our cohort and is a limitation that future research should address.

## 5. Conclusions

This study aimed to investigate paraspinal muscle characteristics in patients with degenerative thoracolumbar kyphosis (DTLK) and lumbar spinal stenosis (LSS), and to identify correlations with thoracolumbar kyphosis (TLK) degree, body mass index (BMI), and lumbar compression index value (LCIV). In patients with DTLK combined with LSS, LCIV was lower than in the control group, and it increased progressively from the upper to the lower lumbar spine. In the control group, paraspinal muscle content increased with age and BMI, and LCIV was higher in males than in females. However, the DTLK group showed no gender difference. LCIV in the DTLK group was more pronounced in patients with increased LL. The degree of TLK was not influenced by BMI but was associated with the content of the paravertebral muscle. For patients with DTLK and LSS, there is a predictable relationship between paraspinal muscle content and the degree of TLK.

## Figures and Tables

**Table 1:** Basic information between DTLK group and control group.

	DTLK Group	Control Group	*p*
Case	126	87	
Gender			0.829
Male	35	23	
Female	91	64	
Age, years	68.0 ± 8.1	68.4 ± 8.3	0.711
BMI, kg/m^2^	26.1 ± 3.6	26.3 ± 3.4	0.745
N: 18.5–24.9	52	35	
OW: 25–29.9	50	39	
OB: ≥30	24	13	
TLK, °	25.8 ± 10.1	6.7 ± 5.4	<0.001
LL, °	36.6 ± 19.1	43.9 ± 11.3	0.001

DTLK: degenerative thoracolumbar kyphosis; BMI: body mass index; N: normal; OW: overweigh; OB: obesity; TLK: thoracolumbar kyphosis; LL: lumbar lordosis.

**Table 2:** Comparisons on LCIV from T12-L5 between DTLK and control groups.

LCIV, mm	DTLK Group	Control Group	*p*
T12-L1	4.4 ± 5.4	9.8 ± 4.4	<0.001
L1-L2	5.4 ± 5.7	10.0 ± 4.4	<0.001
L2-L3	6.7 ± 5.9	9.8 ± 4.4	<0.001
L3-L4	9.2 ± 6.2	10.0 ± 4.4	0.361
L4-L5	13.7 ± 5.6	12.6 ± 5.2	0.202
mLCIV	7.9 ± 5.1	10.4 ± 3.9	<0.001

LCIV: lumbar crossing indentation value; DTLK: degenerative thoracolumbar kyphosis; mLCIV: mean LCIV from T12-L5.

**Table 3:** Comparisons on LCIV between subgroups in terms of gender.

LCIV, mm	DTLK Group			Control Group		
	Male	Female	*p*	Male	Female	*p*
T12-L1	2.4 ± 6.5	5.1 ± 4.9	0.044	11.0 ± 4.5	9.5 ± 4.3	0.198
L1-L2	4.1 ± 7.1	5.8 ± 5.1	0.241	12.1 ± 4.5	9.3 ± 4.2	0.015
L2-L3	7.4 ± 7.3	6.4 ± 5.4	0.527	12.3 ± 4.2	9.0 ± 4.1	0.003
L3-L4	10.1 ± 9.2	8.9 ± 4.9	0.575	12.1 ± 4.6	9.3 ± 4.1	0.014
L4-L5	15.2 ± 8.0	13.2 ± 4.6	0.265	14.9 ± 4.2	11.9 ± 5.4	0.028
mLCIV	7.8 ± 7.0	7.9 ± 4.3	0.979	12.5 ± 3.7	9.8 ± 3.8	0.008

LCIV: lumbar crossing indentation value; DTLK: degenerative thoracolumbar kyphosis; mLCIV: mean LCIV from T12-L5.

**Table 4:** Comparisons on LCIV between subgroups in terms of BMI.

LCIV, mm	DTLK Group			Control Group		
	N	OW	OB	N	OW	OB
T12-L1	2.7 ± 4.1 bb	4.1 ± 6.2 c	7.7 ± 4.2	7.9 ± 4.7 a,bb	10.4 ± 3.7 c	13.2 ± 2.8
L1-L2	3.4 ± 4.3 bb	5.6 ± 6.5	8.2 ± 4.8	8.2 ± 4.9 a,bb	10.6 ± 3.3	12.8 ± 3.9
L2-L3	5.1 ± 5.2 b	7.0 ± 6.6	8.7 ± 5.2	8.0 ± 4.3 a,bb	10.6 ± 3.8	12.3 ± 4.3
L3-L4	8.8 ± 5.4	9.3 ± 7.4	9.8 ± 5.0	8.2 ± 4.0 a,bb	10.8 ± 4.2	12.3 ± 4.6
L4-L5	12.8 ± 5.5	14.0 ± 6.5	14.5 ± 4.0	10.8 ± 5.5 bb	14.1 ± 4.5	13.0 ± 5.2
mLCIV	6.6 ± 4.2 b	8.0 ± 6.0	9.8 ± 4.0	8.6 ± 3.9 aa,bb	11.3 ± 3.3	12.7 ± 3.6

LCIV: lumbar crossing indentation value; BMI: body mass index; DTLK: degenerative thoracolumbar kyphosis; N: normal; OW: overweigh; OB: obesity; mLCIV: mean LCIV from T12-L5. a: statistical difference between N and OW in the same level (*p* < 0.05); aa: statistical difference between N and OW (*p* < 0.01); b: statistical difference between N and OB (*p* < 0.05); bb: statistical difference between N and OB (*p* < 0.01); c: statistical difference between OW and OB (*p* < 0.05).

**Table 5:** Multiple linear regression analysis of mLCIV.

	Coefficient	Unstandardized		Standardized	t	*p* Value
		B	SE	Beta		
DTLK group	Constant	13.749	1.027		13.386	<0.001
	TLK	−0.183	0.036	−0.480	−5.131	<0.001
Control group	Constant	1.016	3.161		0.321	0.749
	BMI	0.356	0.120	0.328	2.970	0.004
All cases	Constant	5.407	2.149		2.516	0.013
	TLK	0.246	0.080	0.215	3.062	0.003
	BMI	−0.127	0.022	−0.413	−5.872	<0.001

DTLK: degenerative thoracolumbar kyphosis; LCIV: lumbar crossing indentation value; SE: standard error; BMI: body mass index. B: unstandardized coefficients, which indicate how much the dependent variable varies with an independent variable when all other independent variables are held constant. Beta: standardized coefficients, which show the strength of the effect that each independent variable has on the dependent variable.

## Data Availability

The data that support the findings of this study are available from our hospital but restrictions apply to the availability of these data, which were used under license for the current study, and so are not publicly available. Data are, however, available from the authors upon reasonable request and with permission of our hospital. Interested parties may contact the first author for further information.
